# Investigation of neuro-inflammatory parameters in a cuprizone induced mouse model of multiple sclerosis

**DOI:** 10.3906/biy-2104-88

**Published:** 2021-10-18

**Authors:** Timucin AVŞAR, Gökçe ÇELİKYAPI ERDEM, Gökhan TERZİOĞLU, Eda TAHİR TURANLI

**Affiliations:** 1 Medical Biology Department, School of Medicine, Bahçeşehir University, İstanbul Turkey; 2 Dr. Orhan Ocalgiray Molecular Biology and Genetics Research Center, İstanbul Technical University, İstanbul Turkey; 3 Department of Biotechnology, Institute of Science, Yeditepe University, İstanbul Turkey; 4 Molecular Biology and Genetics Department, Acıbadem University, İstanbul Turkey

**Keywords:** Cuprizone mouse model, multiple sclerosis, demyelination, cytokines, cellular immune response, oligodendrocyte maturation

## Abstract

Cuprizone, copper chelator, treatment of mouse is a toxic model of multiple sclerosis (MS) in which oligodendrocyte death, demyelination and remyelination can be observed. Understanding T and B cell subset as well as their cytokines involved in MS pathogenesis still requires further scrutiny to better understand immune component of MS. The study presented here, aimed to evaluate relevant cytokines, lymphocytes, and gene expressions profiles during demyelination and remyelination in the cuprizone mouse model of MS. Eighty male C57BL/6J mice fed with 0.2% cuprizone for eight weeks. Cuprizone has been removed from the diet in the following eight weeks. Cuprizone treated and control mice sacrificed biweekly, and corpus callosum of the brain was investigated by staining. Lymphocyte cells of mice analyzed by flow cytometry with CD3e, CD11b, CD19, CD80, CD86, CD4, CD25 and FOXP3 antibodies. IFN-gamma, IL-1alpha, IL-2, IL-5, IL-6, IL-10, IL-17, TNF-alpha cytokines were analyzed in plasma samples. Neuregulin 1 (Nrg1), ciliary neurotrophic factor (Cntf) and C-X-C chemokine receptor type 4 (Cxcr4) gene expressions in corpus callosum sections of the mice brain were quantified. Histochemistry analysis showed that demyelination began at the fourth week of cuprizone administration and total demyelination occurred at the twelfth week in chronic model. Remyelination occurred at the fourth week of following withdrawal of cuprizone from diet. The level of mature and activated T cells, regulatory T cells, T helper cells and mature B cells increased during demyelination and decreased when cuprizone removed from diet. Further, both type 1 and type 2 cytokines together with the proinflammatory cytokines increased. The level of oligodendrocyte maturation and survival genes showed differential gene expression in parallel to that of demyelination and remyelination. In conclusion, for the first-time, involvement of both cellular immune response and antibody response as well as oligodendrocyte maturation and survival factors having role in demyelination and remyelination of cuprizone mouse model of MS have been shown.

## 1. Introduction

Multiple sclerosis (MS) is an immune-mediated, neuro-inflammatory, and neurodegenerative disease of the central nervous system (CNS) with the morphological hallmarks of inflammation, demyelination, and axonal loss (Grigoriadis et al., 2004; Disanto et al., 2010; Lassmann 2013). Although more than a century has passed since the identification of clinical and pathological characteristic of MS, both the etiology and molecular pathogenesis of this disease are not yet conclusively known. The clinical course of MS is highly variable and includes several subtypes and variants (Siva, 2006). Both pathological and immunological studies suggest that different cellular pathways may be active in different MS subtypes (Goldenberg, 2012; Hauser et al., 2013; Avsar et al., 2015).

Several well-characterized experimental animal models of MS have enhanced our understanding of the pathophysiology, such as experimental allergic encephalomyelitis (EAE), toxin-induced demyelination models, and virus-induced models. Among these, EAE has been one of the most frequently studied one, which mirrors inflammatory activity and has helped to understand therapeutic intervention strategies in MS (Robinson et al., 2014). However, EAE model does not directly allow us to explore parameters related to demyelination and remyelination, which are key characteristics of the disease. On the other hand, cuprizone-induced mouse model is commonly used to specifically analyze nonautoimmune mediated demyelination and spontaneous remyelination processes combined with therapeutic effects (Praet et al., 2014).

Cuprizone, (bis–cyclohexanone-oxaldihydrazone) a copper chelator, induces a consistent demyelination of the corpus callosum (CC) to young adult mice, that leads to changes at the cellular and molecular level including oligodendrocyte death, microglial and astrocyte reactivation macrophage accumulation, and T cell differentiations involving T-helper and regulatory T cells (Zhan et al., 2020). When cuprizone is applied for only six weeks, neurodegeneration becomes acute and full recovery is seen after being removed from the diet, while it becomes chronic when administered up to twelve weeks and remyelination is not observed even if it is removed from the diet. Cuprizone-induced model reflects the degeneration of oligodendrocytes rather than direct loss of myelin sheet that provides another line of evidence on the assumption that inflammatory demyelination may not be the only process relating to MS pathogenesis (Acs and Kalman 2012). Furthermore, this approach in the acute model produces a possibility for an assessment of remyelination. It enables to monitor the molecular and cellular changes during the recovery processes.

Cuprizone model also provides opportunity to investigate immune system interference during the formation of demyelination and remyelination because demyelination occurs before the activation of inflammation elements like macrophages / microglia, astrocytes and formation of oligodendrocyte progenitor cell as well, mature oligodendrocytes (Mason et al., 2000; Matsushima and Morell 2001). Therefore, cuprizone model is a still good model to focus on more on the neurodegenerative aspects of MS compared to other animal models of MS (Hochstrasser et al., 2018). 

In this study, we aimed to assess the effects of cuprizone induction on the level of inflammatory cytokines, T and B cell subsets during demyelination and remyelination through the administration of cuprizone for 8 weeks and further 8 weeks for the recovery. Furthermore, during 16 weeks of experiment, the transcript levels of Neuregulin 1 (Nrg1), Ciliary neurotrophic factor (Cntf) and C-X-C chemokine receptor type 4 (Cxcr4) genes, which are important in oligodendrocyte survival, maturation, and migration were analyzed.

## 2. Materials and methods

### 2.1. Mice and cuprizone treatment

Eighty (48 test and 32 control group) C57BL/6J male mice with age of 5 to 6 weeks were used. They were kept together in groups of four mice in 20 cages in standard mouse facility conditions.  Experimental procedures were started after two weeks of acclimatization. Experimental group of mice was provided powder chow containing 0.2% cuprizone (bis-cyclohexanone oxaldihydrazone, Sigma, St. Louis, MO) for 8 weeks as previously described (Kanekiyo et al., 2013). Mouse bait was prepared by mixing 100 g of powder chow with 200 mg cuprizone to obtain 0.2% cuprizone chow mixture; a total of 50 g chow was daily provided for subject group, and excess chow was daily replaced. Control group did not receive cuprizone and they were kept in separate cages. The powder cuprizone was mixed with into ground chow ad libidum in a bowl inside their cages. Tap water was used; water and food levels were checked and refreshed daily. Cuprizone was removed from the diet at the 8th week. The number of animals restricted considering both statistical requirements and ethical issues; therefore, biweekly sacrification of 10 mice (6 cuprizone fed and 4 control mice) were performed for 16 weeks. Experiments were repeated twice. At the first study, 3 subject and 2 control mice were sacrificed for biweekly analysis. At the second study, additional 3 subject and 2 control mice were used. In total, 6 cuprizone treated and 4 untreated (total of 10 mice) for each time point were used. Sacrification was performed under deep anesthesia. Blood sample was removed from the heart of mice via open-heart surgery. All the blood was removed and aliquoted for FACS analyses for both cell surface markers, and plasma was obtained for the cytokine assays. Total brain was removed and embedded into 10% formalin for immunohistochemistry analyses.

All animal procedures were in accordance with the ethical considerations and were approved by the İstanbul University, Cerrahpaşa School of Medicine, Animal Care And Use Committee.

### 2.2. Histochemistry and Staining

The paraffin embedded tissues were sectioned at 5 mm in thickness from the corpus callosum according to the mouse atlas by Sidman et al. (Sidman et al., 1971) For routine histology, the sections were stained with hematoxylin and eosin (H&E) and luxol fast blue periodic acid schiff (LFP-PAS) for myelin sheet observation. Sections were placed in 10 mM citrate buffer (pH = 6) and boiled for 5 min in a microwave. Sections were subsequently incubated (30 min, 37°C) in a buffer containing 5% serum of the host animal. Myelin Basic Protein (MBP) staining was done to assess myelination score by using MBP antibody (12) (Novus, NB600-717), primary antibody reagents were incubated either at 37 °C for 2 h or overnight at 4 °C. Sections were then washed and incubated for 30 min at room temperature with the appropriate secondary Ab conjugated with either Horse Radish Peroxidase (HRP) or Streptavidin visualization. Slides were mounted with Vectashield medium (Vector Laboratories) and visualized by using a light microscope (Leica DMLe, Germany).

Morphology of the tissue and cells were monitored at 10X, 40X and 100X under immersion oil. In order to evaluate the demyelination, three independent researchers observed the slides in a double-blind fashion. Sections were semi quantitatively scored from zero to three as described by Lindner and colleagues (Lindner, et al. 2008). Zero indicated the total demyelination, and three indicated the normal myelination of brain section with no myelin loss. Subsequently, score one indicates the myelination of 1/3, score two; 2/3 of myelin fibers.  

### 2.3. Flow cytometry analyses

Single-cell suspensions of peripheral blood mononuclear cells (PBMC) were prepared, followed by lysis of Red Blood Cells (RBC) in 0.83% NH_4_Cl. Cells were first treated with PBS supplemented with 5% FCS (Fetal Calf Serum). Nonspecific antibody binding was prevented by using 1mg per sample of Fc blocker (BD, Mouse Fc Block, Cat No: 553142) was used by 20 min of incubation. Antibodies against CD3e (17A2), CD11b (M1/70), CD19 (1D3), CD45 (30-F11), CD80 (B7-1, 16-10A1), CD83 (Michel17), CD86 (GL1), CD4 (GK1.5), CD25 (PC61.5) conjugated antibodies (eBioscience) were used for cell surface staining and FOXP3 (JFK-16s) were used for intracellular staining of activated T cells. Cells were fixed and permeabilized by using eBioscienc Foxp3 / Transcription Factor Fixation/Permeabilization concentrate and diluent (E Bioscience, Cat No:00-5521) according to manufacturer’s protocol. First cell pellet including a million cells was incubated with conjugated antibody solution for 15 min at dark and room temperature. Then pellet was washed by centrifugation at 800g for 5 min twice. Then resuspended cell solution was read at FACSCalibur (Becton Dickinson Biosciences). A total of 10,000 events were analyzed for each sample. The lymphocyte gate was defined by forward and side scatters. Antibodies were observed by FITC, PE, and APC channels. (Supplementary Figure). Data were analyzed with CellQuest Software (Becton Dickinson Biosciences).

For cytokine assay, Mouse Th1/Th2 10plex kit (eBioscience, BMS820FF), which is a bead-based analyte detection system, was used with the plasma of mice blood. Assay contains type 1 cytokines, including IL-2, IFN-gamma, IL-12, TNF-beta, and type 2 cytokines including IL-4, IL-5, IL-6, IL-10 and IL-13. Different set of 2 beads sized with 4mm and 5mm are coated with antibodies reacting with corresponding cytokines. Then each analyte to be measured was incubated either with standards or samples. A biotin- conjugated second antibody binding to first antibody attached to the microbeads was used. Streptavidin – Phycoerythrin was added, and the level of cytokines was measured.

### 2.4. Quantitative real time PCR (qPCR)

Corpus callosum (cc) region of the brain was removed according to mouse brain atlas description (Sidman, et al. 1971). RNA was isolated from the cc by RNA isolation kit (Roche High Pure RNA Tissue Kit), and 1mg of RNA obtained. mRNA was converted to complementary DNA (cDNA) by using revert aid first strand cDNA synthesis kit (Roche). Primers for Cxcr4, Cntf and Nrg1 target genes and reference gene Gapdh1 were listed in Supplementary Table. Gene expression levels were calculated by delta delta Ct method.

### 2.5. Statistical analysis

Data were evaluated using unpaired Student’s t-test for histochemistry results, analysis of variance (ANOVA) for FACS and qPCR analysis and expressed as mean ± standard deviation. Myelin scores were compared by using multiple t test comparisons. Statistical analysis was performed using the GraphPrism program package, version 8 (GraphPad Software, San Diego, CA, USA).  

## 3. Results

### 3.1. Eight-week cuprizone administration induced chronic, partly reversible demyelination

**Figure 1:**
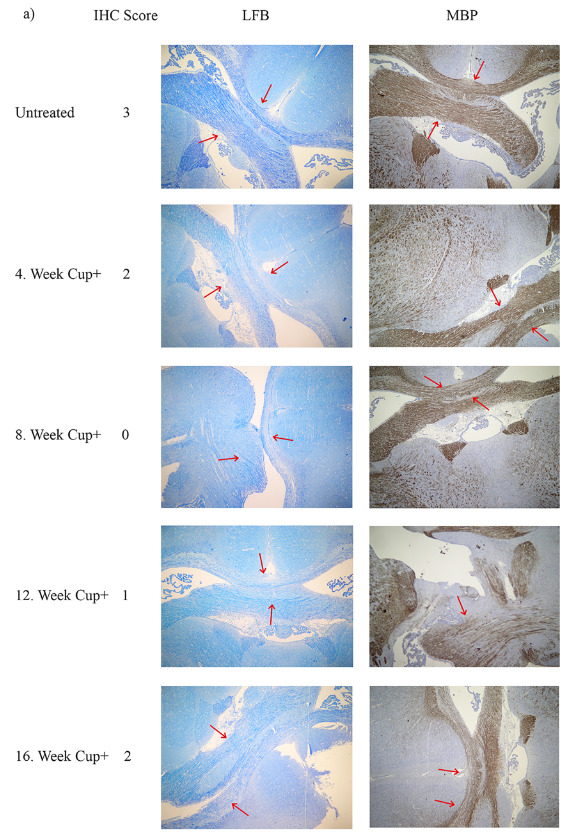

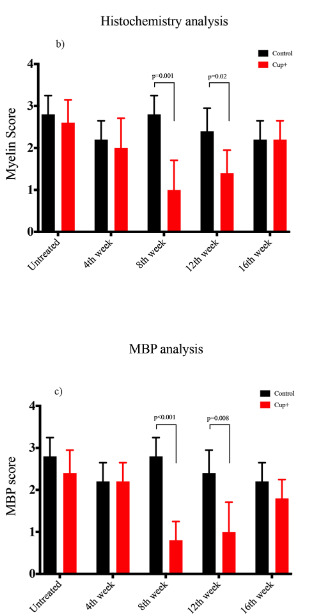
a) Histochemistry analysis showed the total demyelination and remyelination in corpus callosum section of the mice brain treated with 8 weeks of cuprizone administration and following 8 weeks of withdrawal from the diet. Scores indicated myelination score which is given by two independent pathologists. 3: no effect on myelination, +2: initial demyelination, +1: partial demyelination, +0: total demyelination. Luxol Fast blue staining, MBP: Myelin basic protein staining. Arrows indicate corpus callosum region. b) Mean myelin scores of cuprizone treated and untreated groups. Demyelination significantly differed at the 8th week (p = 0.001) and 12th week of treatment (p = 0.02) c) Mean myelin scores by using anti-MBP antibody staining of cuprizone treated and untreated groups. Demyelination significantly differed at the 8th week (p < 0.001) and the 12th week of the treatment (p = 0.008). “Control” indicates mice group without cuprizone, and “Cup+” indicates cuprizone treated mice group.

Degree of demyelination in brain was evaluated in the CC by LFB-PAS and MBP immune - stained sections. Based on the LFB-PAS stained CC sections, degree of demyelination was scored regarding the dissemination of CC integrity. Semiquantitative scoring of LFB-PAS staining showed that demyelination was maximum (2.3 ± 0.4) at 8. week for the cuprizone fed mice, whereas control mice showed no demyelination. Following the removal of cuprizone from the diet at the 8th week, mice showed remyelination, starting from the 14th week to the 16th week, showing better amelioration each week. Anti-MBP staining showed the level of MBP density at the CC sections. The level of MBP staining was parallel with that of demyelination. The level of anti-MBP staining was 1.2 ± 0.5 at 8th week and 2.6 ± 0. 4 at 1th week, indicating the remyelination after the cuprizone removal from the diet (Figure 1).

### 3.2. T and B cells subsets levels increased during demyelination and decreased after cuprizone treatment

**Figure 2:**
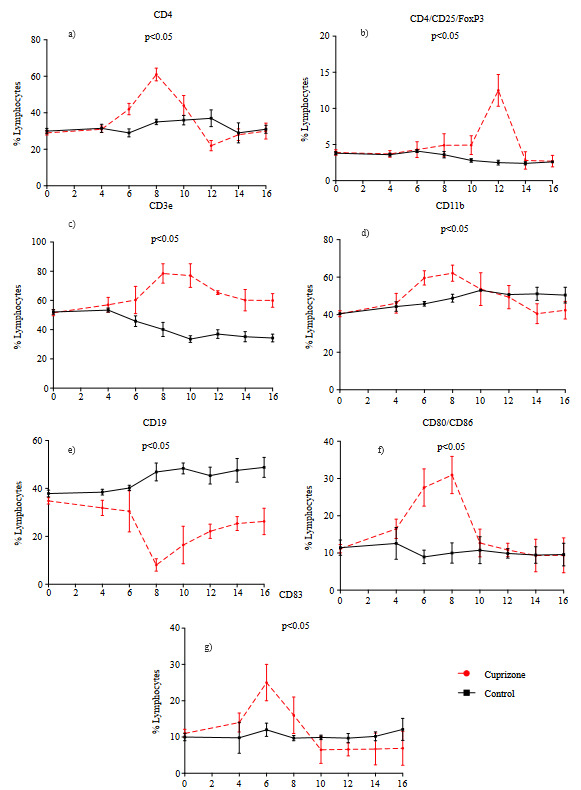
Flow cytometry analysis of cuprizone treated and untreated mice. Data were derived from peripheral blood mononuclear cells (PBMS), and analysis have been done at lymphocyte gate. a) CD4, b) CD4/CD25/FOXP3 triple positive, c) CD3e, d) CD11b, e) CD19, f) CD80/CD86 double positive, g)CD83 positive cells. Y-axis indicates total lymphocytes gated, and X-axis indicates the weeks. Six subjects, four control mice were used for each week.

The level of CD4 positive (CD4+) T and/or T helper cells increased to 61 ± 3.5% at 8th week of cuprizone treatment and decreased to 30 ± 4.4% at 16th week, whereas control mice did not show significant change (p < 0.05) (Figure 2a). Antiinflammatory regulatory T cell level (Treg cells), as indicated by CD4 + / CD25 + and FoxP3 + cells, increased to 12.5±2.2 by the 10th week and decreased to 2.8 ± 1.2% at 14th week, whereas control mice did not show significant change (p < 0.05) (Figure 2b). The level of mature T cells in the immune system was assessed by evaluation of CD3e (17A2) positive (CD3e+) cells. In cuprizone-induced mice, the CD3e+ T cell level increased to 78.4 ± 6.7% at 8th week and decreased thereafter with the removal of cuprizone from diet (p < 0.05) (Figure 2c). Macrophage antigen-1 (Mac-1), CD11b, positive cells that shows the frequency of active macrophages, were increased to 62.1 ± 4.3% by week 8 and then decreased to 42.4 ± 4.7% by week 16. Active macrophage cell frequency significantly increased in response to cuprizone treatment (p < 0.05), while control mice did not show significant change (Figure 2d). 

B cell involvement in the chronic demyelination model significantly altered when compared to control mice. B lymphocyte (CD19+) cell level decreased to 8.1 ± 2.6% by week 8 and when cuprizone was withdrawn from the diet, increased to 26.25 ± 5.5%, (p < 0.05), whereas control mice did not show any significant change over 16 weeks remained in average of 44.2 ± 2.8% (Figure 2e). On the other hand, the activated / mature B lymphocyte (CD80/CD86 + cells) and antigen presenting cell (CD83+ cell) level increased to 31.0 ± 5.0% by the 8th week (Figure 2f) and 25.0 ± 5.0% by the 6th week (Figure 2g), respectively, in response to cuprizone (p < 0.05). Investigation on leukocyte levels showed that T helper and T regulatory cell levels increased during demyelination and decreased after cuprizone was removed from the diet. B cell markers and costimulatory B cells showed the B cell response during the demyelination period.

### 3.3. Cuprizone treatment induced expression of various inflammatory cells and cytokines as immune response 

**Figure 3:**
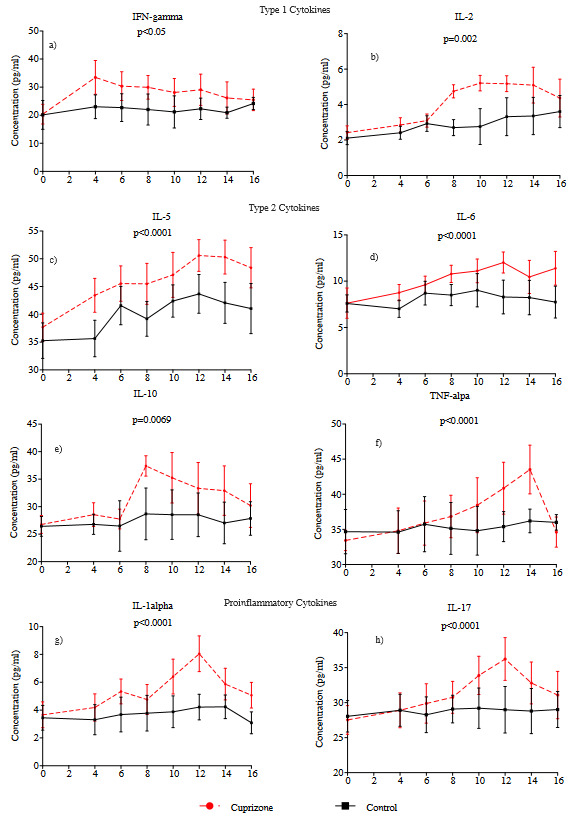
Cytokine levels of cuprizone treated and untreated mice. Data were obtained from plasma of mice blood. Y-axis indicates total cytokines gated, and X-axis indicates the weeks. Six subjects, four control mice were used for each week.

Eight weeks of cuprizone treatment leading to demyelination triggered alterations in the level of various pro- and anti-inflammatory cytokines at different times, indicating a hypothetical sequence of immune cell recruitment and activation. T helper and T regulatory cell levels secreting pro- and anti-inflammatory cytokines increased during demyelination and decreased after cuprizone withdrawal from the diet. Type I cytokines IFN-gamma and IL-2 significantly increased in response to cuprizone treatment indicating cytokine based immune response. IFN-gamma level increased to 30.0 ± 4.2% at the 6th and 8th week of cuprizone treatment and decreased to 25.5 ± 3.9 over 16 weeks (p < 0.05) (Figure 3a). IL-2 increased to 5.2 ± 0.4% in subject group, whereas it did not significantly change in controls (p = 0.0022) (Figure 3b). Type 2 cytokines IL-5, IL-6 Il-10 and TNF-alpha significantly changed between week 8 to week 14. IL-5 (50.6 ± 2.9%, p < 0.0001) and IL-6 (12.0 ± 1.5%, p < 0.0001) were maximum at week 12 (Figure 3c, 3d). IL-10 was maximum (37.4 ± 1.8%, p = 0.0069) at week 10 (Figure 3e), and TNF-alpha was maximum (43.5 ± 3.5, p < 0.0001) at week 14 (Figure 3f). Proinflammatory cytokines IL-1alpha (8.1 ± 1.3, p < 0.0001), and IL-17 (36.2 ± 3.1, p < 0.0001) levels were maximum at week 12 and significantly differed from that of control mice (Figure 3g and 3h). 

Cytokine analysis showed that the level of proinflammatory cytokines was increased as initial response to cuprizone treatment during demyelination period, whereas antiinflammatory cytokines’ level increased thereafter at the late stage of chronic demyelination. Involvement of both Th1 and Th2 cytokines at different weeks of cuprizone treatment indicated that complex interaction of various inflammatory cells and cytokines were increased as immune response to cuprizone. However, further studies are required on both cellular and functional studies.

### 3.4. Cuprizone treatment affected the oligodendrocyte maturation and survival associated gene expression

**Figure 4:**
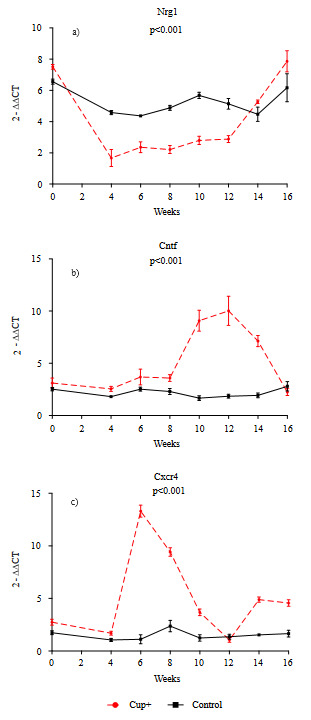
Gene expression analysis for oligodendrocyte maturation and survival factors. Data were obtained from mRNA of total corpus callosum (cc) homogenate. Y-axis indicates relative gene expression by means of delta delta Ct analysis with regard to GAPDH gene level. X-axis indicates the weeks. Three subjects, two control mice were used for each week. “Control” indicates mice group without cuprizone, and “Cup+” indicates cuprizone treated mice group.

Neuregulin 1 (Nrg1) is epidermal growth factor family protein and necessary for development of oligodendrocytes (Mei and Xiong 2008). The level of Nrg1 gene expression decreased significantly as an initial response to cuprizone treatment between week 0 and week 4 and remained the same between weeks 4 and 12 until the cuprizone was removed, then increased to baseline, while the control group showed no significant difference for 16 weeks (p < 0.001) (Figure 4a). Ciliary neurotrophic factor (Cntf) is a survival factor for both neurons and oligodendrocytes (Stankoff et al., 2002). Cntf expression decreased between week 0 and week 4, then remained the same until week 8, but it then increased to a maximum by week 12 and decreased immediately (p < 0.001) (Figure 4b). C-X-C chemokine receptor type 4 (Cxcr4) is a chemokine that is essential for the mobilization of hematopoietic cells and oligodendrocytes. Cxcr4 expression showed dramatic changes between the 4th and 12th weeks. Cxcr4 level increased by week 6 and then gradually decreased and remained the same through week 12 (p < 0.001) (Figure 4c). All gene expression results indicated chronic demyelination pattern, which dramatic expression level changes occurred between the 4th and 12th weeks. 

## 4. Discussion

Cuprizone treatment of mice induces a highly reproducible and consistent demyelination of distinct brain regions, such as the corpus callosum (CC), which represents the most frequently investigated white matter part in this animal model (Hochstrasser et al., 2018; Zhan et al., 2020). Five to six weeks of cuprizone treatment induces almost completely demyelinated CC, which leads to acute demyelination. When cuprizone is removed from the diet in the weeks following acute demyelination, spontaneous remyelination follows. In contrast, when the use of cuprizone is extended (12 weeks or longer), remyelination is severely restricted and chronic demyelination develops. In the late stages of acute demyelination, spontaneous remyelination partially occurs but fails under an ongoing cuprizone treatment. (Skripuletz et al., 2008; Torkildsen et al., 2008). In this study, eight weeks of 0.3% cuprizone treatment triggered chronic demyelination in CC, and only partial remyelination was achieved with the withdrawal of cuprizone from the diet.

MS previously considered as Th1-mediated autoimmune disease (Jadidi‐Niaragh and Mirshafiey, 2011; Lassmann and Ransohoff, 2004). This concept was developed based on MS pathological and immunological studies obtained from both human samples and the experimental autoimmune encephalomyelitis (EAE) model. However, more recently a wide variety of inconsistent data with the CD4-Th1 driven hypothesis has emerged and supported the idea that other immunological mechanisms including IL-23 cytokines and Th-17 cells, as well as neurodegenerative components within the target tissue may also contribute to the initiation, spread, and modification of disease (Jadidi‐Niaragh and Mirshafiey, 2011). Current evidence on CD4 + autoreactive T cells supports this concept as a central factor for the autoimmune pathogenesis of MS (Sospedra and Martin, 2005). The cuprizone model allows us to analyze the levels of these cell types and their cytokines independently of the CD4 + T cell-based immunological induction of MS. In this study, eight weeks of cuprizone treatment increased the level of CD4 + T helper cells until cuprizone was removed from the diet and decreased immediately. However, the level of regulatory T (Treg) cells (CD4 + / CD25 + / FoxP3) increased significantly until week 12 of the experiment. Treg cells are naturally occurring suppressor cells that can prevent reactivity to both self and nonself antigens. They can both enhance and suppress the activity of T cells (Lee, 2018). In this study, the levels of Treg cells were increased by week 12 in response to cuprizone treatment, then decreased by week 16. Although cuprizone was removed from the diet after 8 weeks; the level of regulatory T cells increased over the next 4 weeks. This may be the result of an increased immune response that is regulated and suppressed by Treg cells. Results of Treg studies in MS patients are conflicting. The level of FoxP3 protein and mRNA in CD4+/CD25+ T cells decreased in MS patients compared to healthy controls (Fritzsching et al., 2011). However, there are other studies indicating the decreased or aberrantly changed regulatory T cell levels in MS patients (Venken et al., 2008; Viglietta et al., 2004). Regarding the conflicting results in the patient studies and the cuprizone model, we say that the role of Treg cells in MS pathogenesis changes depending on the dynamic and varying nature of the disease. In addition, cuprizone model is only a model of MS showing the partial characteristics of the disease on mice. It may not be reflecting the T cell profile of disease on mice.

The level of CD3e + cells that function to activate cytotoxic T cells and helper T cells increased significantly at 8 weeks, decreased slightly 10 weeks after the removal of the cuprizone and then significantly decreased. CD3e results showed consistency with Treg and CD4+ T cell levels. CD11b is known as macrophage-1 antigen (Mac-1) and expressed on various types of leukocytes including monocytes, granulocytes, and macrophages. CD11b+ cell level increased significantly at the 6th and 8th weeks of cuprizone treatment. The level of CD3e + and CD11b + cells in the blood indicates the onset of an acute inflammation at the 6th week of treatment followed by an increase in the level of T helper and Treg cells and suppressing active inflammation.

The role of B cells in MS is diverse as both antibody secreting plasma cells and antibody producing cells. They can act as both anti- and proinflammatory cells in MS pathogenesis (Lehmann-Horn et al., 2013). In addition, B cell activation markers within the CSF of MS patients predict the conversion between clinical subtypes of MS and are associated to clinical parameters (Disanto et al., 2012). CD19 is a B cell lymphocyte antigen and expressed in all B lineage cells (Rich, et al. 2012). Although many studies showed increased B cell count in MS patients and an increase in the EAE model, to our knowledge, studies demonstrating the role of B cells in the cuprizone-induced demyelination and remyelination model are limited. In this study, the level of CD19+ cells significantly increased in cuprizone treated mice. After cuprizone removal, B cell level decreased. Furthermore, the level of CD80 and CD86 double positive cells, expressed on B cells, antigen presenting cells, macrophages and dendritic cells, increased in response to cuprizone treatment. CD80/CD86+ cells indicate the induced Th1 cell responses via binding CD28 on T cells. CD80/CD86 increase causes activation, proliferation, and increase in T cell cytokine secretion (Li et al., 2016). It was also shown that the level of CD80/CD86 cells decreased in response to interferon β-1b therapy (Jensen et al., 2010). CD83 level was measured to assess antigen presenting cell activation (Li et al., 2019). Dendritic cells are critically involved in initiating primary immune response and T cell dependent antibodies (Vinuesa et al., 2010). In the cuprizone model, the level of CD83+ cells significantly increased at 6th week and dramatically decreased thereafter. The results may show that active inflammation in the cuprizone model starts at earlier weeks with proliferation of helper T cells as well as B cells and then regulated by Treg cells.

Cytokine profile in serum sample of mice correlated with cell surface receptor analysis. IFN-Gamma which is a pro-inflammatory cytokine secreted by activated T lymphocytes, dramatically increased at the 4 week of treatment and slightly decreased thereafter. Previous study by Gao X. et al also indicated an elevated IFN-gamma expression in response to cuprizone treatment (Gao et al., 2000). Furthermore, they also showed that low level IFN-gamma expressing transgenic mice did not show extensive demyelination unlike the cuprizone treated wild type model. IL-17, IL-1alpha and TNF-alpha function as pro-inflammatory cytokines that responds to the invasion of the immune system and production of inflammation. In cuprizone model, the level of IL-17 and IL1-alpha increased after 12 weeks of experiment, whereas TNF-alpha increased at 14th week. Cytokine analysis showed that the level of pro-inflammatory cytokines increased as a response to cuprizone diet during demyelination period, but reached the maximum level at different weeks, showing a cascade of expression, whereas antiinflammatory cytokines’ level increased later at the second stage of chronic demyelination. IL-10 and IL-6 are the antiinflammatory cytokines that down regulates the expression of Th1 cytokines. As expected during remyelination, the level of IL-10 and IL-6 are significantly differed compared to control mice. 

Nrg1 (Neuregulin 1), Cntf (Ciliary Neurotophic Factor), Cxcr4 (C-X-C Chemokine Receptor 4) gene expressions were analyzed in this study as they play important roles in oligodendrocyte survival, maturation and oligodendrocyte precursor cell migration to the demyelination site. In particular, Cxcr4 is an important receptor for the chemokine Cxcl12, and the binding of Cxcr4-Cxcl12 is important for oligodendrocyte progenitor cell migration in the brain. Cxcr4 is expressed by hematopoietic stem cells and Cxcl12 is expressed by astrocytes and microglia in demyelination site. Previous studies show that Cxcl12 expression levels decrease with cuprizone treatment (Li et al., 2012; Sanadgol et al., 2017). Nrg1 and Cntf are important factors for oligodendrocyte maturation and survival (Stankoff et al., 2002; Taveggia et al., 2008). Cntf is a polypeptide hormone, which affect the central nervous system. Cntf is expressed by neurons and microglia in CNS. Cntf injection to develop rat optic nerve increases cell survival by 80%. Nrg1 is a protein and have 6 alternative splicing form. Nrg1 is expressed on neurons’ cell surface and bind to ErbB receptors on oligodendrocytes. Nrg1 promote the oligodendrocyte survival through ErbB receptors on oligodendrocytes (Newbern and Birchmeier, 2010). However, Nrg1 is not an essential factor for central nervous system myelination. Nrg1 knock-out mice central nervous system can be myelinated (Velanac et al., 2012). Nrg1 is an essential factor for Schwann cell survival. Schwann cells are myelin cells in peripheral nervous system. Therefore, Nrg1 is an important and essential factor for peripheral nervous system myelination. If gene expression changes of Nrg1 and Cntf in the cuprizone model can be detected, their role in remyelination and how they cause chronic demyelination can be demonstrated, so that new treatments for MS and alternative strategies for remyelination can be developed. Our results indicated that, Nrg1 gene expression significantly decreases after 4 weeks of cuprizone treatment (p < 0.001), but it almost did not change in 4th–12th weeks period (4th–10th week p < 0.001). After 12 weeks, Nrg1 expression increased to baseline with the removal of cuprizone from the diet. Cntf decreased within the first 4 weeks of cuprizone treatment (p < 0.05) but increased in 8–12 weeks of cuprizone treatment (6th–8th weeks and p < 0.05, 8–10 weeks and p < 0.001). After 12 weeks, Cntf expression increased to baseline with the removal of cuprizone from the diet. Similarly, Cxcr4 expression decreased by the 4th week (p <0.05) and reached its maximum level at 6th week (p < 0.05). It then decreased over a 6 to 12 week period (p <0.005), reaching a minimum at week 12. At the 12th week, cuprizone was removed from the feed, and the expression level of Cxcr4 increased to baseline after 4 weeks (p < 0.001). Only Cntf gene expression level decreased after the 12th week, Nrg1, Cxcr4 level increase after the 12th week. Cxcr4 expression change pattern correlate with acute demyelination to chronic demyelination transition. Cxcr4, Cntf, Nrg1 gene expressions turn into the initial level after the withdrawal of cuprizone from the chow. Although three gene expressions change upon cuprizone treatment, solely is not sufficient to claim that oligodendrocyte maturation and survival is changed; it was only hypothesized that oligodendrocyte cell death and further survival/maturation changes in MS pathogenesis may be associated with the Cxcr4, Cntf and Nrg1 gene expressions. However, further studies with detailed cellular and functional analysis are required. 

In conclusion, we provide new evidences that the expression profile of surface receptors and cytokine profile of cuprizone treated mice for 8 week showed similarity of MS disease course. Th1 dependent immune response and B cell costimulation increased during the demyelination and decreased during remyelination, which is the following 8-week period after cuprizone removal. In light of our results, further investigations addressing specific questions on the mechanisms underlying the etiology and pathogenesis of MS will be needed. The cascade of proinflammatory and antiinflammatory cytokines needed to be questioned. Such research should include immunological markers, clinical as well as response to therapeutic intervention parameters. 

## Informed consent

This study was approved by the Animal Use and Ethical Committee of İstanbul University, Cerrahpaşa School of Medicine. 

## References

[ref1] (2012). Pathogenesis of multiple sclerosis: what can we learn from the cuprizone model. In: Autoimmunity.

[ref2] (2015). CSF proteomics identifies specific and shared pathways for multiple sclerosis clinical subtypes. PloS one 10:e0122045..

[ref3] (2010). Heterogeneity in multiple sclerosis: scratching the surface of a complex disease. Autoimmune Diseases.

[ref4] (2012). The evidence for a role of B cells in multiple sclerosis. Neurology.

[ref5] (2011). Intracerebral human regulatory T cells: analysis of CD4+ CD25+ FOXP3+ T cells in brain lesions and cerebrospinal fluid of multiple sclerosis patients. PloS one.

[ref6] (2000). Interferon-γ protects against cuprizone-induced demyelination. Molecular and Cellular Neuroscience.

[ref7] (2012). Multiple sclerosis review. Pharmacy and Therapeutics.

[ref8] (2004). Axonal damage in multiple sclerosis: a complex issue in a complex disease. Clinical Neurology and Neurosurgery.

[ref9] (2013). Multiple sclerosis: prospects and promise. Annals of Neurology.

[ref10] (2018). Do pre-clinical multiple sclerosis models allow us to measure neurodegeneration and clinical progression?. Expert Review of Neurotherapeutics.

[ref11] (2011). Th17 cell, the new player of neuroinflammatory process in multiple sclerosis. Scandinavian Journal of Immunology.

[ref12] (2010). Immunoglobulin-like transcript 3, an inhibitor of T cell activation, is reduced on blood monocytes during multiple sclerosis relapses and is induced by interferon β-1b. Multiple Sclerosis Journal.

[ref13] (2013). Loss of branched O-mannosyl glycans in astrocytes accelerates remyelination. The Journal of Neuroscience.

[ref14] (2013). Pathology and disease mechanisms in different stages of multiple sclerosis. Journal of the neurological sciences 333.

[ref15] (2004). The CD4–Th1 model for multiple sclerosis: a crucial re-appraisal. Trends in Immunology.

[ref16] (2018). The balance of Th17 versus Treg cells in autoimmunity. International Journal of Molecular Sciences.

[ref17] (2013). Targeting B cells in the treatment of multiple sclerosis: recent advances and remaining challenges. Therapeutic Advances in Neurological Disorders.

[ref18] (2016). CD80 and CD86 knockdown in dendritic cells regulates Th1/Th2 cytokine production in asthmatic mice. Experimental and Therapeutic Medicine.

[ref19] (2012). Chemokine CXCL12 in neurodegenerative diseases: an SOS signal for stem cell-based repair. Trends in Neurosciences.

[ref20] (2019). CD83: Activation marker for antigen presenting cells and its therapeutic potential. Frontiers in Immunology.

[ref21] (2008). Sequential myelin protein expression during remyelination reveals fast and efficient repair after central nervous system demyelination. Neuropathology and Applied Neurobiology.

[ref22] (2000). Mature oligodendrocyte apoptosis precedes IGF-1 production and oligodendrocyte progenitor accumulation and differentiation during demyelination/remyelination. Journal of Neuroscience Research.

[ref23] (2001). The Neurotoxicant, Cuprizone, as a Model to Study Demyelination and Remyelination in the Central Nervous System. Brain Pathology.

[ref24] (2008). Neuregulin 1 in neural development, synaptic plasticity and schizophrenia. Nature Reviews Neuroscience.

[ref25] (2010). Nrg1/ErbB signaling networks in Schwann cell development and myelination. In: Seminars in Cell & Developmental Biology. Elsevier.

[ref26] (2014). Cellular and molecular neuropathology of the cuprizone mouse model: Clinical relevance for multiple sclerosis. Neuroscience & Biobehavioral Reviews.

[ref27] (2012). Clinical Immunology E-Book: Principles and Practice. Elsevier Health Sciences.

[ref28] (2014). The experimental autoimmune encephalomyelitis (EAE) model of MS: utility for understanding disease pathophysiology and treatment. Handbook of Clinical Neurology.

[ref29] (2017). Neuroprotective effects of ellagic acid on cuprizone-induced acute demyelination through limitation of microgliosis, adjustment of CXCL12/IL-17/IL-11 axis and restriction of mature oligodendrocytes apoptosis. Pharmaceutical Biology.

[ref30] (1971). Atlas of the mouse brain and spinal cord. Harvard University Press.

[ref31] (2006). The spectrum of multiple sclerosis and treatment decisions. Clinical Neurology and Neurosurgery.

[ref32] (2008). Cortical demyelination is prominent in the murine cuprizone model and is strain-dependent. The American Journal of Pathology.

[ref33] (2005). Immunology of multiple sclerosis. Annual Review of Immunology.

[ref34] (2002). Ciliary neurotrophic factor (CNTF) enhances myelin formation: a novel role for CNTF and CNTF-related molecules. Journal of Neuroscience.

[ref35] (2008). Type III neuregulin‐1 promotes oligodendrocyte myelination. Glia.

[ref36] (2008). The cuprizone model for demyelination. Acta neurologica Scandinavica Supplementum.

[ref37] (2012). Bace1 processing of NRG1 type III produces a myelin‐inducing signal but is not essential for the stimulation of myelination. Glia.

[ref38] (2008). Natural naive CD4+ CD25+ CD127low regulatory T cell (Treg) development and function are disturbed in multiple sclerosis patients: recovery of memory Treg homeostasis during disease progression. The Journal of Immunology.

[ref39] (2004). Loss of functional suppression by CD4+ CD25+ regulatory T cells in patients with multiple sclerosis. The Journal of Experimental Medicine.

[ref40] (2010). T cells and follicular dendritic cells in germinal center B‐cell formation and selection. Immunological Reviews.

[ref41] (2020). The cuprizone model: dos and do nots. Cells.

